# Bringing together components of the fly renal system

**DOI:** 10.1016/j.gde.2009.08.006

**Published:** 2009-10

**Authors:** Barry Denholm, Helen Skaer

**Affiliations:** Department of Zoology, Downing Street, Cambridge CB2 3EJ, UK

## Abstract

The function of all animal excretory systems is to rid the body of toxins and to maintain homeostatic balance. Although excretory organs in diverse animal species appear superficially different they are often built on two common principals: filtration and tubular secretion/reabsorbtion. The *Drosophila* excretory system is composed of filtration nephrocytes and Malpighian (renal) tubules. Here we review recent molecular genetic data on the development and differentiation of nephrocytes and renal tubules. We focus in particular on the molecular mechanisms that underpin key cell and tissue behaviours during morphogenesis, drawing parallels with other species where they exist. Finally we assess the implications of patterned tissue differentiation for the subsequent regulation of renal function. These studies highlight the continuing usefulness of the fly to provide fundamental insights into the complexities of organ formation.

## Introduction

The functional role of excretory systems is fundamentally similar in all multicellular animals; namely the elimination of metabolic and foreign toxins and homeostasis, the maintenance of ionic, acid–base and water balance. The predominant metabolic toxins are nitrogenous waste products, which are excreted as ammonia, urates or other derivatives such as guanin. Some foreign toxins taken up from the environment can be excreted through the activity of transporters that have evolved to eliminate these compounds in the urine (e.g. the renal tubules of some herbivorous insects that ingest ouabain express cardiac glycoside transporters) [1]. Novel foreign toxins can only be eliminated by default, either by passively leaking into the urine or through active filtration of the body fluids. Homeostasis is effected in the majority of animals by tubular epithelia in which a primary urine, isosmotic with the blood/body fluids is modified by the activity of transporters to retrieve valuable ions and molecules, to balance H^+^/HCO_3_ and to regulate water loss. To satisfy these requirements renal systems are commonly made up of a filtration system and simple tubular elements, either bathed in body fluids or in close contact with blood vessels.

In the majority of excretory organs ultrafiltration by specialised filtration cells produces a primary filtrate, which is modified as it passes along an epithelial tube, before being excreted. This is true of nephrons in the vertebrate kidney [2] and for a variety of invertebrate excretory systems, such as the protonephridial system of the flatworm [3], or arthropod segmental glands such as the maxillary and antennal glands of crustaceans and the coxal glands of Chelicerates [4–9]. In these systems the site of filtration is physically linked to the modifying tubule. However in some arthropods primary urine is actively secreted by the tubule cells themselves and the site of filtration, by specialised cells called nephrocytes, is physically distinct from the renal tubules.

An excellent and comprehensive survey of animal excretory systems has recently been published [10]. In this review we focus on *Drosophila* nephrocytes and renal tubules, discussing the molecular basis for aspects of their morphogenesis and differentiation and pinpointing those that reveal wider parallels with other renal systems.

## Ultrafiltration by nephrocytes

**Figure 1:**
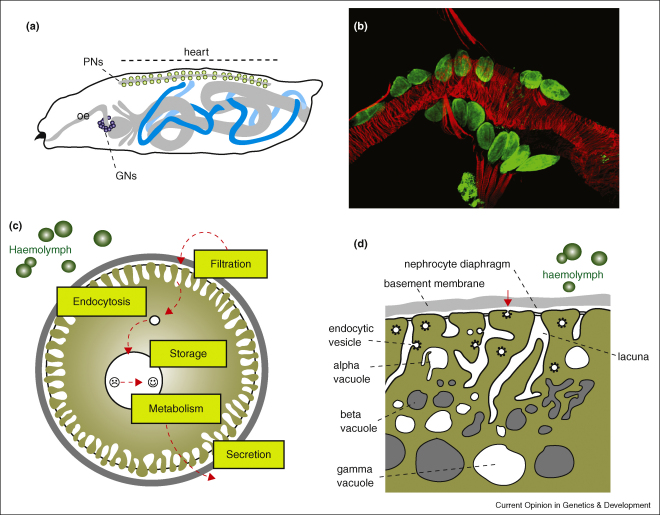
**(a)** Cartoon to show the mature arrangement of pericardial (PNs, green circles) and garland (GNs, purple circles) nephrocytes, and Malpighian tubules (blue) in the late embryo/larva. The heart (linear grey structure) runs along the dorsal (upper) side of the animal. The alimentary canal (including oesophagus, oe) is depicted in grey. **(b)** Third instar larval pericardial nephrocytes (green, anti-Sns. Smaller stained cells, bottom are fat body) lined up on either side of the heart (red, phalloidin stain for actin. Alary muscles also appear red). Anterior to the left. **(c)** Cartoon of a nephrocyte. Haemolyph is filtered across basement membrane (grey outer circle) and nephrocyte diaphragm (black line linking nephrocyte foot processes) entering the lacunae, from where it is endocytosed (small white circle). Endocytosed material is stored and/or metabolised (large white circle). The stored and/or metabolised material can be secreted back into the haemolymph. Haemocytes (green circles). **(d)** Cartoon to show detail of the nephrocyte cortical region. Salient features including basement membrane, nephrocyte diaphragm and lacunae are labelled. Endocytosis from lacunae walls and foot process tips (red arrow) are shown. Vacuoles of different type (alpha, beta and gamma) are located within the cell. Haemocytes (green circles).

Nephrocytes were first described by Kowalevsky [11] as ‘storage kidneys’ because of their capacity to sequester and store materials from the haemolymph. Two nephrocyte types are found in *Drosophila*: pericardial nephrocytes, two rows flanking the heart, and garland nephrocytes, a necklace of cells surrounding the oesophagus (Figure 1a,b). A third type not found in *Drosophila*, the so-called disseminated nephrocytes, are scattered singly throughout the fat body and other tissues in some insect species [12]. Nephrocytes are derived from the embryonic mesoderm and many persist through metamorphosis into adult life [13,14]. Pericardial nephrocytes are derived from the dorsal-most, cardiac mesoderm that also gives rises to the heart and lymph gland (the source of adult blood cells or haemocytes) [15,16]. By the end of embryogenesis a heterogeneous population of 120 pericardial cells (based on differential gene expression) can be found surrounding the heart (Figure 1a [13]). However, larval and adult pericardial nephrocytes number only about 40 (our unpublished data) [14,17]. The significance of this reduction and the destiny of the majority of cells that disappear are unknown (some *eve* positive pericardial cells form the adult ‘wing heart’ [18]). The garland nephrocytes develop from a subset of procephalic or head mesoderm cells, the ‘posterior late secondary head mesoderm’ (lSHMp), which gives rise to garland nephrocytes and embryonic/larval blood cells. The lSHMp delaminates during embryonic stages 9–11 and garland nephrocyte precursors, initially arranged as two bilateral clusters, migrate medially and dorsally where they fuse to form a crescent of cells under the oesophagus (Figure 1a [19]).

The intimate ontogenetic relationship between vascular cells, blood cells and excretory cells is a widespread feature in animal development, common to both coelomate invertebrates and vertebrates. Furthermore, a conserved network of regulatory genes and signalling pathways underpins their development in diverse animal species (e.g. see [16,20,21]). Drawing examples from living invertebrates and vertebrates, Hartenstein and Mandel [22] argue that these developmental relationships were a feature of the ancestral bilateria. They propose that cells within the mesothelium — the mesodermal lining of the coelom — acquired vascular and excretory functions, and that these cells were the likely forerunner of the specialised vascular, blood and excretory cells found in extant animals.

Nephrocytes primarily function to regulate haemolymph composition by filtration and filtrate endocytosis. Extensive infoldings of the plasma membrane generate a network of ‘labyrinthine channels’ or ‘lacunae’ flanked by nephrocyte foot processes. The entrance to these channels takes the form of narrow slits ∼30 nm in width, spanned by single or double filaments that form a specialised filter known as the nephrocyte diaphragm (Figure 1c,d) [15,23–25,26^••^,27]. Each nephrocyte is enveloped in a negatively charged basement membrane so that haemolymph is filtered across basement membrane and the nephrocyte diaphragm and is endocytosed from the lacunae. This barrier effectively excludes particles larger than ∼10–12 nm in diameter [23,25]. We found that a 10 kDa fluorescently labelled dextran (10.6 nm diameter) passes through the filter whereas a larger 500 kDa dextran (93 nm diameter) does not [26^••^]. The intrinsic negative charge of the basement membrane additionally acts as a charge-selective filter preventing passage of molecules with high negative charge [25].

The filtration barrier in the glomerulus of the vertebrate kidney is of strikingly similar appearance and function. This is formed by specialised epithelial cells called podocytes that send out interdigitating foot processes to enwrap the glomerular capillaries. These processes are separated by 30–50 nm slit pores spanned by the slit diaphragm, which, together with a negatively charged basement membrane, forms a size-selective and charge-selective filtration barrier. Glomerular permeability for a given particle is thus determined by its size and charge, albumin for example is a negatively charged 67 kDa (7.2 nm diameter) protein that does not pass through the barrier. Experiments exploring slit diaphragm permeability in the fish pronephros similarly showed passage of smaller (10 kDa dextran) but not larger (500 kDa dextran) compounds [28]. In the kidney ultrafiltration is driven by blood pressure. Without a closed circulatory system, it has been suggested that local pressure differences generated by the movement of adjacent peristaltic tissues (heart and oesophagus) drive ultrafiltration and irrigation of the lacunae in the nephrocyte [25].

The molecular make-up of the insect nephrocyte diaphragm has recently been found to share a high degree of similarity with the slit diaphragm [26^••^,27]. The molecular core of both diaphragms are two immunoglobulin-superfamily (Ig-SF) proteins, nephrin/NEPH1 in the slit diaphragm and the *Drosophila* orthologues, Sticks and stones (Sns)/Dumbfounded (Duf) in the nephrocyte diaphragm. The extracellular domains of these proteins make homotypic and heterotypic interactions to form the barrier itself. In their absence, such as in the human disease congenital nephrotic syndrome of the Finnish type (NPHS1, caused by genetic defects in nephrin) or in fly mutants for *sns* or *duf*, the diaphragm is completely missing and filtration capabilities of the cell are severely compromised [26^••^,29]. In NPHS1 patients blood proteins leak into the urine (proteinuria), resulting in kidney failure and death if left untreated [30]. Vertebrate nephrin and NEPH1 form a multi-protein complex at the slit diaphragm with CD2-associated protein (CD2AP) and zonula occludens-1 (ZO-1), binding to nephrin and NEPH1, respectively and possibly anchoring them to the actin cytoskeleton, and podocin, a hairpin-like protein of the stomatin family involved in formation of membrane microdomains and lipid raft-associated processes [31–36]. Fly orthologues of these proteins are expressed in nephrocytes and form a complex that closely mirrors the molecular interactions of the vertebrate complex, demonstrating that nephrocyte-diaphragms and slit-diaphragms, in addition to the anatomical and functional similarities described above, are strikingly related at the molecular level. These similarities make it tempting to speculate that nephrocytes and podocytes are homologous cell types; a hypothesis that would be strengthened if the filtration diaphragms of other excretory organs, such as the flame bulb filtration slits in flatworms or the end sac filtration cells in arthropod segmental organs, were also based around an orthologous Ig domain molecular complex.

Nephrocytes are prodigiously active in endocytosis. They appear to sequester colloidal and soluble macromolecules, but do not take up particulate matter [37^••^]. The majority of endocytosis in the nephrocyte takes place after filtration from the lacunae, although endocytosis of non-filtered haemolymph also takes place from the foot process tips to a lesser extent (Figure 1d). It is possible that there is a qualitative difference in endocytosis because vesicle size differs between the two regions [23,25,38]. There appear to be at least two uptake mechanisms macropinocytosis — for the uptake of colloidal material, and micropinocytosis — for the uptake of soluble macromolecules. The molecular mechanisms underpinning endocytosis in nephrocytes are beginning to be understood [37^••^]. Micropinocytosis is dependent on dynamin — a GTPase involved in the scission of newly formed endocytic vesicles [37^••^,38]. By contrast, macropinocytosis is dynamin independent. Instead, the colloidal-uptake pathway requires the function of *Drosophila* Rudhira, a cytoplasmic WD40-domain protein of unknown molecular function that is expressed in larval nephrocytes [17,37^••^]. Whether these different endocytic pathways are spatially segregated to tip versus lacunal membranes has not been investigated. Intracellular recognition, destination, sorting and ultimate fate of endocytosed material also remains obscure. Nephrocytes are certainly capable of long-term storage [11], but there is also evidence that some material is metabolised [39,40] and recycled by exocytosis (Figure 1c).

The architectural integrity of the cortical lacunae requires that endocytosis and exocytosis be held in balance. If this balance is disrupted the size and distribution of the lacunal membranes are severely altered. For example, when endocytosis is blocked using the dynamin temperature-sensitive allele (*shibire*^*ts*^) the lacunae become more labrinthine, indicating an expansion of cell surface area [38]. The kinetics of this change is particularly impressive; the channels lengthen up to six times (from ∼0.5 μm to ∼2–3 μm) within 20 min. By contrast, reduction in biosynthetic exocytic membrane traffic by the expression of a dominant negative Rab11, results in complete loss of lacunae [41]. Thus the nephrocyte lacunal membranes are in constant flux — with vigorous endocytosis sustained by equally dynamic membrane replenishment.

## Urine secretion and modification by renal tubules

**Figure 2:**
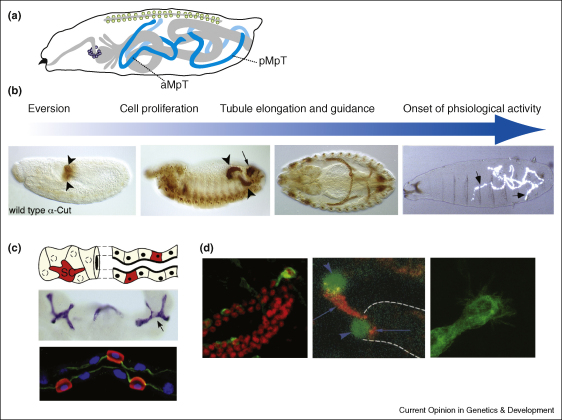
**(a)** Cartoon to show the mature arrangement of the nephrocytes (hollow circles) and Malpighian tubules (blue) in the larva (late embryo/larva). Anterior (aMpT) and posterior (pMpT) are labelled. **(b)** Sequence of developmental events underlying renal tubule maturation. Panels show embryos stained for Cut protein at stages 10, 12, 14 and shown in polarised light stage 17. Arrowheads show the developing tubules, the small arrow the posterior spiracle (also Cut-positive) and large arrows precipitates of uric acid in the mature tubule lumen. **(c)** Principal (no colour) and stellate (red in cartoon and lower panel, blue in central panel) cells in the distal, secretory region of adult (central) and 3rd instar larval tubules. As they integrate SCs become polarised (nuclei in blue, actin in green in lower panel), only developing a stellate morphology in the adult (arrow, central panel). **(d)** Tubule tip cells have a prominent morphology, secrete the EGF ligand sSpitz and show dynamic filipodial activity during tubule elongation. First panel stained for Cut (red) and CD8-GFP (green). Second panel ase-nLacZ line stained for β-Gal (green, arrowheads) and Rhomboid (red, arrows) showing both the tip cell and its sibling. Third panel CD8-GFP line showing the tip cell with many filopodial membrane extensions.

The tubular elements of the *Drosophila* renal system are the four Malpighian tubules (Figure 2a), which are laid down during embryogenesis, grow through larval life without further cell division and persist through metamorphosis into the adult. Recent studies suggest that there is some cell renewal from a stem cell population in the adult tubules [42]. The tubules are ectodermal in origin, budding out during embryogenesis from the hindgut anlage close to its junction with the midgut. These primordial cells divide between two and five times, after which further tubule growth depends on cell rearrangement, enlargement and flattening. The tubules elongate as cells rearrange, extending in a stereotypical pattern through the body cavity to achieve reproducible positions in the mature embryo. Cell differentiation becomes evident through the onset of excretory activity before the end of embryogenesis and is first visible when transported urates precipitate as uric acid in the tubule lumen (Figure 2b). As these aspects of tubule development have recently been reviewed [43], we focus here on two features of tubule morphogenesis; the incorporation of an additional, mesenchymal population of cells into the tubule epithelium and guided tubule extension.

The mature tubules are a single cell-layered epithelium, made up of secretory cells in the distal regions (furthest from the ureters, which connect each pair of tubules to the hindgut) and cells with a largely reabsorbtive function more proximally [44]. There are two secretory cell types that produce primary urine; the principal cells, which transport cations (largely K^+^) into the lumen by proton/cation exchangers [45], and stellate cells, the site of Cl^−^ conductance and aquaporin-mediated water movement [46,47] (Figure 2c). The principal cells derive from the ectodermal primordial buds, while stellate cells (SCs) derive from a mesenchymal population of mesodermal cells, which integrate into the tubules as they elongate [48,49^••^].

Stellate cells express the closely related transcription factors Teashirt and Tiptop [50] as well as Hibris (Hbs), another Ig domain Nephrin homologue [49^••^] (Figure 2c). Teashirt and Tiptop appear to act redundantly in stellate cells; in single mutants for either gene, the number of stellate cells integrated into the tubules remains unaltered (Hu and Skaer, unpubl.). However, Hibris is required for the normal integration of SCs. *hbs*^*361*^*/Df(2R)14* mutants have little *hbs* function and develop tubules with ∼60% of the wild-type SC number, dying as late larvae or young adults with defective excretory function [49^••^]. SC numbers are similarly reduced in the absence of both roughest (*rst*) and *duf* (68% in *Df(1)w67k30*) but are wild-type in *rst*^*6*^ mutants, in which the cytoplasmic domain of Rst is truncated. These data suggest that the reduction in SC number is likely to result from the loss of *duf* (Dix and Skaer, unpubl.). Ig domain proteins interact during myogenesis, signalling through cytoplasmic factors to alter gene expression and cell behaviours (reviewed in [51]). Two factors known to act downstream of Rst/Duf in muscle founder cells to bring about cytoskeletal changes in a Rac-dependent manner also influence tubule development [52^•^]. These authors report that in mutants affecting the function of *myoblast city* (*mbc*) or of a *rolling pebbles* isoform (*rols6*) the number of SCs in mature tubules is ‘slightly’ reduced. Indeed we have found a reduction to 52% of the wild-type SC number in amorphic *mbc*^*C1*^ mutants (Dix and Skaer, unpubl.). The muscle-specific Rols isoform, Rols7, interacts with Duf through TPR-repeats, which are conserved in Rols6 [52^•^,53]. It is thus tempting to speculate that the molecular interactions, which during myogenesis regulate cell fusion, might in the tubules lead to cell intercalation. However only the expression of Rols6 (in PCs [52^•^]) and Hbs (in SCs [49^••^]) have so far been demonstrated in tubules, so that we do not yet know whether, and if so how, Hbs might interact with Duf and its downstream effectors to bring about these tubule-specific cell behaviours.

As the tubules elongate they extend in a remarkably regular fashion, one pair projecting forwards into the thorax and the other running backwards on either side of the hindgut before each crosses it close to the posterior (Figure 2b). These morphogenetic movements produce extended tubules capable of widespread sampling of the body fluids, an important feature for efficient toxin clearance in an animal with an open circulatory system. Strikingly the tips of the mature tubules contact specific tissues; the two posterior tip cells contacting nerves that run up the visceral mesoderm on either side of the hindgut [54] and the anterior tip cells contact alary muscles between abdominal segments 3 and 4 (Weavers and Skaer, unpubl.). The regularity of outgrowth suggests pathfinding by signalling between ‘guidepost’ tissues and tubule cells. Consistent with this idea, tubule outgrowth is perturbed when TGF-β signalling is defective; in mutants for the receptors *punt* and *thick veins* and for the pathway-induced transcriptional regulator *schnurri* the anterior tubules fail to extend forwards but remain coiled close to their point of eversion from the hindgut [55]. The TGF-β ligand, Decapentaplegic (Dpp), is expressed in the dorsal epidermis (reviewed in [56]) and also in a ring of midgut visceral mesoderm [57]. In Ubx^9.22^ mutants, in which the midgut expression of Dpp is lost [58], or if a dominant negative Tkv receptor is expressed in the tubules, the anterior pair fail to project forwards (C Hooley, PhD thesis, Cambridge University; Bunt *et al.*, unpublished data). A similar tubule misrouting phenotype was reported for mutations in *mbc* and *rols6* (in addition to reduced SC number [52^•^]). As *rols6* and *mbc* are expressed in the developing midgut [59,60], it is possible that defective tubule morphogenesis results from failure of the Dpp guidance cue in this tissue. While Dpp appears to regulate the forward extension of the anterior tubules, multiple further cues are likely to control other aspects of tubule outgrowth, including the final tip cell contacts. Tip cells extend dynamic filopodial extensions, apparently sampling the environment through which they move (Figure 2d). The signals that guide them and their responses to these cues will help us to understand how visceral tissues adopt their final three-dimensional shapes and positions.

## Coordinating nephrocyte and tubule activities

In any excretory system that involves waste removal by filtration and primary urine modification the rates of filtration, secretion and reabsorption must be coordinately regulated. Where the sites of filtration and urine production are physically separate this might not seem necessary but insect nephrocytes are thought to act as temporary storage kidneys, taking up toxins but releasing them with or without modification to be excreted passively through the paracellular pathway between tubule cells [11,61–63]. If this were the case, controlled release by nephrocytes at times of maximal diuresis would increase the efficiency of toxin clearance. How might such coordination be achieved? Global haemolymph-borne diuretic regulators control the secretion of primary urine in PCs and SCs, activating at least three pathways (reviewed in [64]). If nephrocytes express receptors for these factors, it is easy to envisage coordinate regulation. Alternatively nephrocytes might be regulated by local circuits; the garland cells by gut peristalsis, reflecting food uptake and metabolic activity, and the pericardial cells by heart rate, another indicator of physiological load. It is provocative to find that tubule tip cells, which regulate aspects of tubule development through their expression of the ligands Delta and EGF (Figure 2d and [54,65]), lie in close proximity to the gut (hindgut visceral nerve/muscle) and heart (alary muscles). Might these two physically distinct renal components achieve coordinated activity through their physical relationships to other tissues and to each other?

## References and recommended reading

Papers of particular interest, published within the period of review, have been highlighted as:

• of special interest
•• of outstanding interest
